# Supplementary material for: Differential Co-expression and Regulatory Network Analysis Uncover the Relapse Factor and Mechanism of T Cell Acute Leukemia
Source: Mol Ther Nucleic Acids. 2018 May 29;12:184–94. doi: 10.1016/j.omtn.2018.05.003 (PMC6023839; doi:10.1016/j.omtn.2018.05.003)
Supplement: Document S1. Figures S1–S3 [file mmc1.pdf]

OMTN, Volume *12*

Supplemental Information

Differential Co-expression and Regulatory Network Analysis Uncover the Relapse Factor and Mechanism of T Cell Acute Leukemia

Mei Luo, Qiong Zhang, Mengxuan Xia, Feifei Hu, Zhaowu Ma, Zehua Chen, and An-Yuan Guo

Supplemental Figures:

Figure S1 The miRNA-TF-gene regulatory network for relapse/remission gene sets.

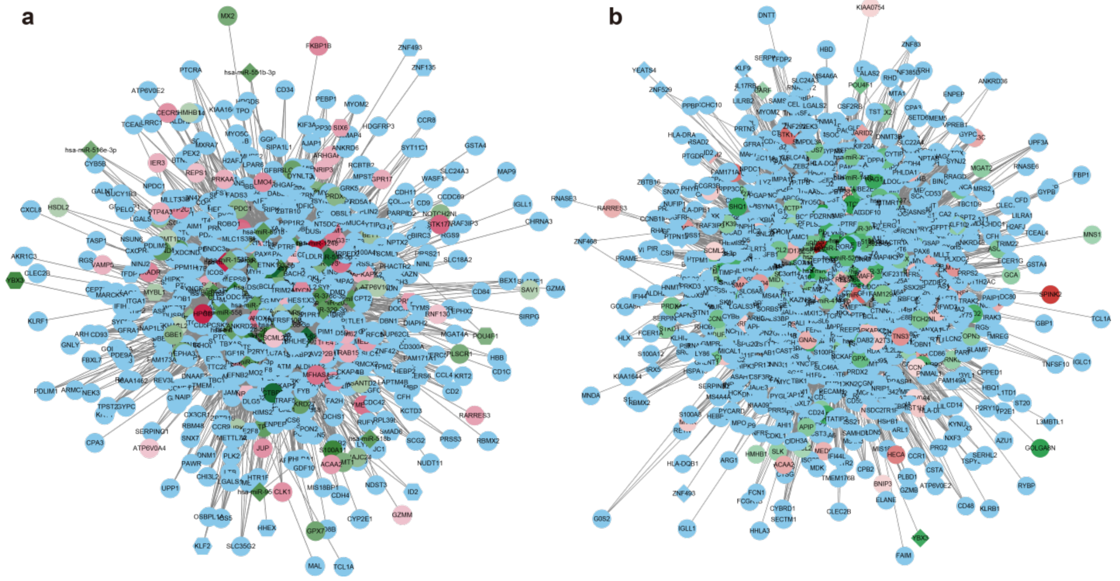

Figure S1 The miRNA-TF-gene regulatory network for relapse/remission gene sets. Hexagon: miRNA; Diamond: transcriptional factor; Ellipse: gene. The red shows up-regulation in relapse, the green represents down-regulation and the blue represents non-DEGs but co-expression. a) The miRNA-TF-gene regulatory network for relapse gene sets. b) The miRNA-TF-gene regulatory network for remission gene sets.

Figure S2 The miRNA-TF-gene regulatory network for DEGs in relapse.

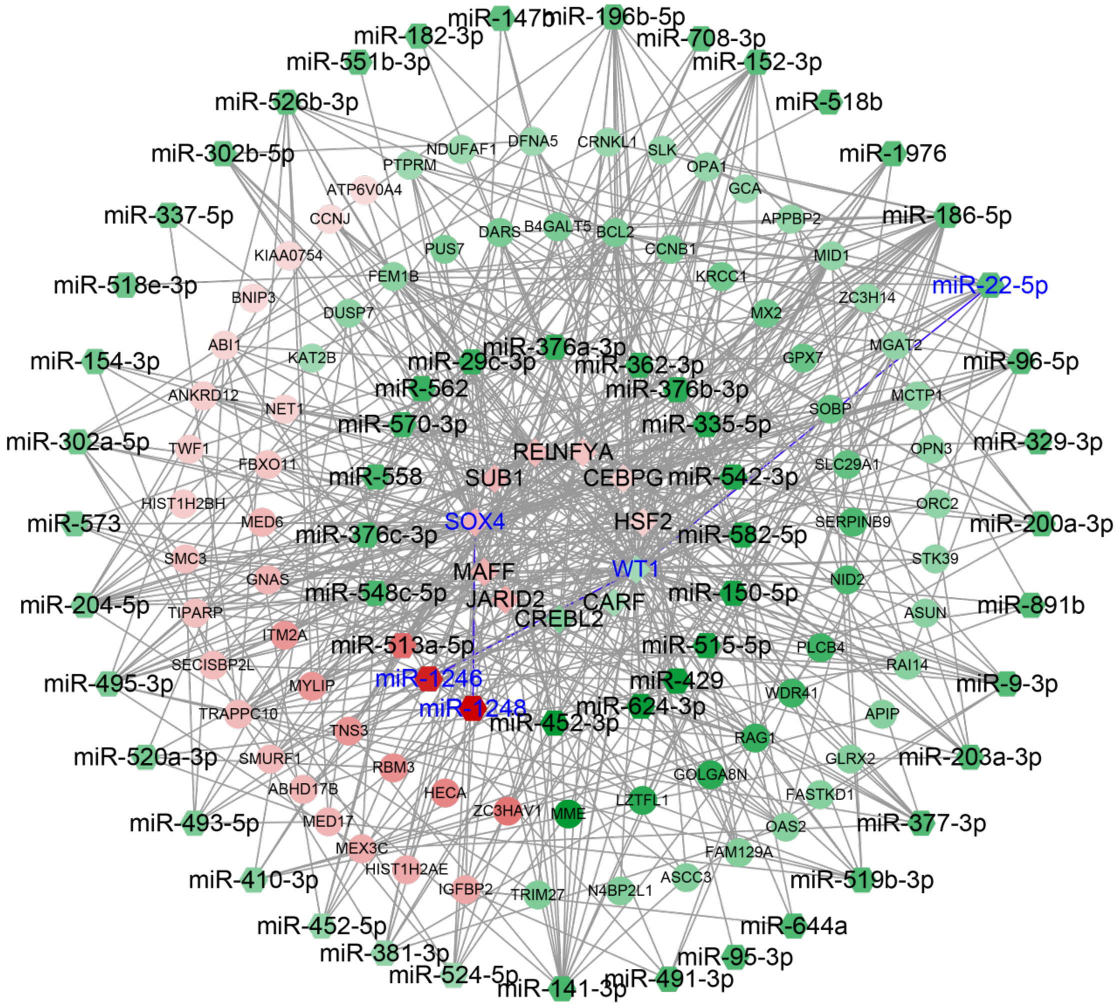

Figure S2 The miRNA-TF-gene regulatory network for DEGs in relapse. Hexagon: miRNA; Diamond: transcriptional factor; Ellipse: gene. The red shows up-regulation in relapse, the green represents down-regulation.

Figure S3 DFS analysis of MFHAS1 and the optimization NOTCH2_siRNA.

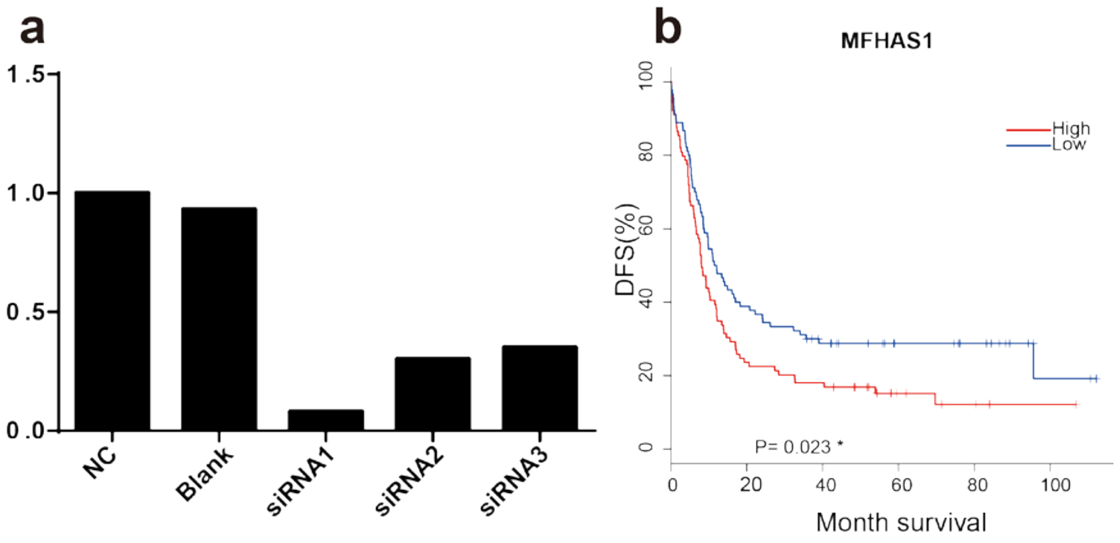

Figure S3 DFS analysis of MFHAS1 and the optimization NOTCH2_siRNA. a) The expression of NOCTH2 transfection with NOTCH2_siRNAs. b) Disease-free survival (DFS) curve of *MFHAS1* in LAML TCGA cohort.
