# Supplementary material for: Differential Co-expression and Regulatory Network Analysis Uncover the Relapse Factor and Mechanism of T Cell Acute Leukemia
Source: Mol Ther Nucleic Acids. 2018 May 29;12:184–94. doi: 10.1016/j.omtn.2018.05.003 (PMC6023839; doi:10.1016/j.omtn.2018.05.003)
Supplement: Document S3. Article plus Supplemental Information [file mmc3.pdf]

Molecular Therapy
Nucleic Acids
Original Article

AMERICAN SOCIETY of GENE & CELL THERAPY

# Differential Co-expression and Regulatory Network Analysis Uncover the Relapse Factor and Mechanism of T Cell Acute Leukemia

Mei Luo,[1,4] Qiong Zhang,[1,4] Mengxuan Xia,[1] Feifei Hu,[1] Zhaowu Ma,[2] Zehua Chen,[3] and An-Yuan Guo[1]

[1]Department of Bioinformatics and Systems Biology, Key Laboratory of Molecular Biophysics of the Ministry of Education, College of Life Science and Technology, Huazhong University of Science and Technology, Wuhan 430074, China; [2]Laboratory of Neuronal Network and Brain Diseases Modulation, School of Medicine, Yangtze University, Jingzhou, Hubei 434023, China; [3]Joint Laboratory for the Research of Pharmaceutics-Huazhong University of Science and Technology and Infinitus, Wuhan, China

**The pediatric T cell acute lymphoblastic leukemia (T-ALL) still remains a cancer with worst prognosis for high recurrence. Massive studies were conducted for the leukemia relapse based on diagnosis and relapse paired samples. However, the initially diagnostic samples may contain the relapse information and mechanism, which were rarely studied. In this study, we collected mRNA and microRNA (miRNA) data from initially diagnosed pediatric T-ALL samples with their relapse or remission status after treatment. Integrated differential co-expression and miRNA-transcription factor (TF)-gene regulatory network analyses were used to reveal the possible relapse mechanisms for pediatric T-ALL. We detected miR-1246/1248 and *NOTCH2* served as key nodes in the relapse network, and they combined with TF *WT1*/*SOX4*/*REL* to form regulatory modules that influence the progress of T-ALL. A regulatory loop miR-429-*MYCN*-*MFHAS1* was found potentially associated with the remission of T-ALL. Furthermore, we proved miR-1246/1248 combined with *NOTCH2* could promote cell proliferation in the T-ALL cell line by experiments. Meanwhile, analysis based on the miRNA-drug relationships demonstrated that drugs 5-fluorouracil, ascorbate, and trastuzumab targeting miR-1246 could serve as potential supplements for the standard therapy. In conclusion, our findings revealed the potential molecular mechanisms of T-ALL relapse by the combination of co-expression and regulatory network, and they provide preliminary clues for precise treatment of T-ALL patients.**

## INTRODUCTION

T cell acute lymphoblastic leukemia (T-ALL), which accounts for 10%–15% of childhood and 25% of adult ALL cases,[1] is an aggressive hematologic neoplasm mainly caused by the malignant transformation of T-lymphocyte progenitors and the accumulation of genomic lesions in T cell development.[2] Although the therapeutic outcome of pediatric T-ALL has improved in recent decades (70%–75% long-term event-free survival rates),[3] it still remains a subgroup of cancers with the worst prognosis, and nearly 20% of child T-ALL patients still suffer relapse and cannot be salvaged by standard therapies.[1,4] Thus, systematically investigating the mechanism of relapse and identifying prognostic biomarkers for pediatric T-ALL will be helpful to clinical diagnosis and therapy.

Previous studies have focused on the genetic and epigenetic abnormalities in pediatric T-ALL and identified some driver events, such as *NOTCH1* mutation, DNA methylation, and leukemia-initiating cell escape.[5,6] Some genes (e.g., *CFLAR* and *BTG3*) have been considered as prognosis markers for the relapse of pediatric T-ALL.[7] Moreover, multiple significant biological pathways have been reported playing vital roles in the recurrence of T-ALL, such as PI3K/AKT and JAK/STAT pathways, which are involved in the proliferation and survival of the leukemia cells.[8,9] However, rare research investigated the difference of transcriptome profiling between the relapse and remission specimens at the initial status (before treatment), which may provide new insights for the precise treatment of pediatric T-ALL. Additionally, microRNAs (miRNAs) as important post-transcriptional regulators play crucial roles in multiple biological processes and diseases, and some related databases are built for further studies.[10,11] Some miRNAs were reported to serve as biomarkers to predict the prognosis of child T-ALL, such as miR-181 and miR-451.[12,13] Meanwhile, transcription factors (TFs) as regulators of gene expression may promote or inhibit the progression of T-ALL, such as *KLF4* and *TAL1*/*SCL*.[14,15] Although some factors were discovered to be associated with the relapse of pediatric T-ALL, the detailed molecular mechanisms have not been investigated so far.

Weighted gene correlation networks and differential co-expression analysis, such as Weighted Gene Co-expression Network Analysis

Received 15 November 2017; accepted 4 May 2018;
https://doi.org/10.1016/j.omtn.2018.05.003.

[4]These authors contributed equally to this work.

**Correspondence:** An-Yuan Guo, Department of Bioinformatics and Systems Biology, College of Life Science and Technology, Huazhong University of Science and Technology, 1037 Luoyu Road, Wuhan 430074, China.
**E-mail:** guoay@hust.edu.cn

 © 2018 The Author(s).
This is an open access article under the CC BY license (http://creativecommons.org/licenses/by/4.0/).

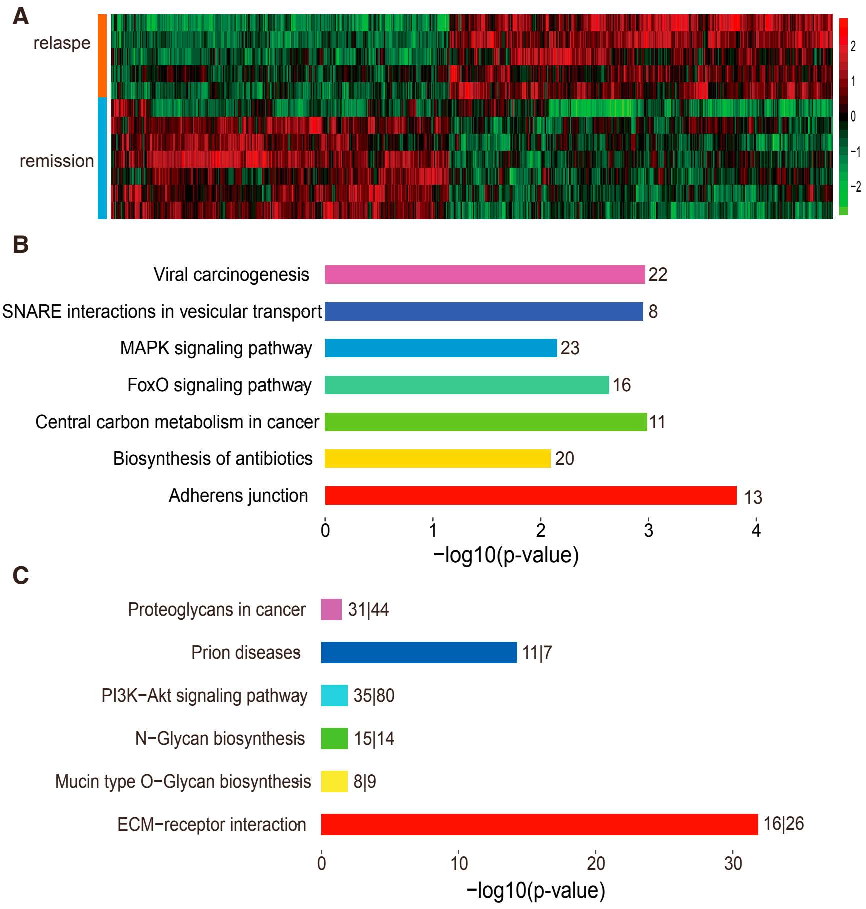


**Figure 1. The Function Enrichment Analysis**

(A) The clustered heatmap of all DEGs in relapse and remission. Relatively higher expression is shown in red and lower expression is in green. (B) KEGG enrichment of DEGs by DAVID database (p value < 0.01). Number beside bar, counts enriched in KEGG pathway. (C) KEGG enrichment of DEMs by DIANA-miRPath (p value < 0.05). Number beside bar, targets of DEMs enriched in KEGG pathway; m/n, DEMs/targets.

(WGCNA) and coXpress, can help to identify important gene modules in specific biological processes and diseases through functional module detection.[16,17] The gene expression regulatory networks, especially the miRNA-TF-gene co-regulatory networks based on feedback loops (FBLs) and feedforward loops (FFLs), have been applied to reveal the inner relationship among regulatory factors, gene expression, and diseases.[18] We have applied the miRNA-TF-gene network and identified the miR-19-*CYLD-NFKB* module in the development of T-ALL.[19] Thus, the combination co-expression and gene regulatory network analysis may better explain the association of gene modules and diseases.

In this study, based on gene and miRNA expression data from initially diagnosed patients with follow-up relapse or remission status after standard Berlin-Frankfurt-Münster (BFM) treatment, we performed gene co-expression and miRNA-TF-gene network analysis to reveal potential molecular mechanisms underlying the relapse of pediatric T-ALL. We identified relapse and remission-specific regulatory networks and potential key modules, which were associated with the relapse of pediatric T-ALL. Meanwhile, our study highlights the advantages of regulatory networks combined with functional module analysis in exploring the mechanism of diseases.

## RESULTS

### Identification of Differentially Expressed Genes and miRNAs

To detect important alterations of transcriptome profiling relevant to the relapse of child T-ALL, we performed differential expression analysis of genes and miRNAs on relapse and remission samples before treatment. In the comparison of relapse versus remission group, we found 832 genes (442 upregulated and 390 downregulated) and 61 miRNAs (5 upregulated and 56 downregulated) were significantly differentially expressed (DEGs and DEMs).

Hierarchical clustering of these DEGs excellently segregated the relapse and remission samples into discrete categories (Figure 1A), which indicated these DEGs can differentiate the relapse and remission specimens well. Functional enrichment results demonstrated the DEGs were relevant to the processes of leukemia and cancer, such as Central carbon metabolism in cancer, MAPK-signaling pathway, and FoxO-signaling pathway (Figure 1B). The MAPK signaling pathway regulates various cellular processes, including cell proliferation, survival, differentiation, and migration.[20] Among these DEGs, *KRAS* contributes to the leukemogenic transformation,[21] and PTEN mutation occurs in 11.1% pediatric T-ALL patients as a tumor suppressor.[22] Moreover, among those DEGs, 324 genes have stable expression profiles, which meant the expressions of all specimens in one experimental condition (relapse or remission) were higher or lower than the other (Table S1). These stable genes may be the pivotal markers of T-ALL relapse (Figure 2). For instance, we focused on the top five DEGs with stable upregulated and downregulated profiling across groups. The *CTBP2*, as a co-repressor in the NOTCH-signaling pathway, was stably downregulated in the relapse group (Figure 2); *SERPINB9*, as a granzyme B inhibitor significantly downregulated in main subtypes of pediatric ALL,[23] was stably downregulated in the relapse group and consistent with the previous study.

Meanwhile, target genes of the 56 downregulated DEMs were assigned to 6 Kyoto Encyclopedia of Genes and Genomes (KEGG) pathways (p value < 0.05; Figure 1C), such as extracellular matrix (ECM)-receptor interaction (p value < 0.001) and PI3K-Akt-signaling

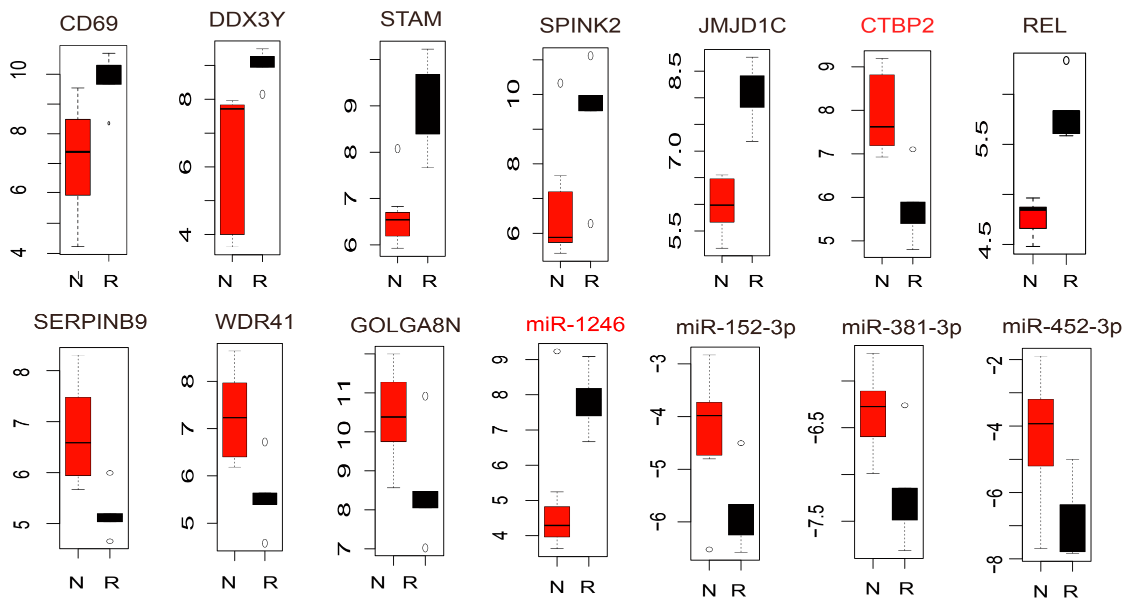


**Figure 2. The Expression of DEGs and DEMs with Stable Pattern**

Expression levels of top 5 upregulated and top 5 downregulated DEGs and all DEMs with stable expression profile in relapse and remission samples. R, relapse; N, remission.

pathway (p = 0.012). ECM-receptor interaction pathway, which plays important roles in cell migration and proliferation,[24] was identified as the most significant pathway in our results. PI3K-Akt-signaling pathway, reported as an oncogenic pathway in T-ALL,[25] was enriched by the most targets of DEMs in our results. These results implied the DEMs may contribute to the relapse of pediatric T-ALL through diminishing the inhibition effects of oncogenes, which thereby activate carcinogenic pathways in T-ALL. Furthermore, we identified four DEMs (miR-1246, miR-152-3p, miR-381-3p, and miR-452-5p) with stable expression profiles (Figure 2), and miR-152 was reported to be associated with poor clinical outcome for ALL in infants.[26] The miR-1246, which was highly expressed and stably upregulated in the relapse group (fold change = 7.37, p value = 0.003), may regulate the progress of T-ALL (Figure 2). Hence, DEGs and DEMs with stable expression profiles may be potential markers to predict the relapse of T-ALL and to explore the underlying molecular mechanism.

### Detection of the Relapse- and Remission-Specific Gene Sets in Pediatric T-ALL

To systematically investigate the potential functional modules in relapse and remission conditions, we performed differential co-expression module analysis for genes with coefficient of variation (CV) > 10% by combining coXpress with WGCNA methods. The detailed work flow is shown in Figure 3. Using coXpress, 7 significantly differential co-expression modules with 742 genes were detected in relapse condition at the given significant level ($p < 0.05$), while 8 modules containing 374 genes were identified in remission condition (Table 1). On the other hand, 22 modules with 1,455 genes were detected by WGCNA in all samples (Table S2). Notably, 10 of the 22 modules were strongly associated with immunity, and 8 modules were related to cell proliferation (Figure 4A). The results demonstrated the modules by WGCNA may be strongly associated with the progression of T-ALL.

To obtain the comprehensive gene sets relevant to relapse/remission, we merged the relapse/remission-specific modules with WGCNA modules through hypergeometric test, respectively ($p < 0.05$; Figures 3 and 4A). As a result, 6 WGCNA modules were assigned to the relapse gene set, and 4 of them were mainly involved in the immunity/cell proliferation processes. Another 6 WGCNA modules related to immunity and cell proliferation were merged with the remission gene set (Figure 4A). Finally, we obtained 1,002 genes associated with the relapse condition and 583 genes related to remission, respectively. Furthermore, the relapse/remission-specific gene sets shared some common pathways related to cancer and hematopoietic system, such as transcriptional misregulation in cancer, hematopoietic cell lineage, and pathways in cancer (Figure 4B). Targeting the PI3K-Akt-signaling pathway could strengthen remission of acute myeloid leukemia (AML) through enhancing the drug sensitivity,[27] and this pathway here was specifically enriched by the remission gene set. The pathway "osteoclast differentiation" peculiarly enriched by the relapse gene set may indicate the tumor cells hijack the bone-remodeling process and create a fertile microenvironment for tumor growth.[28]

### Molecular Regulatory Network Analysis of Modules in Relapse and Remission Conditions

To reveal the mechanism of gene expression regulation in the relapse and remission of pediatric T-ALL, we built miRNA-TF-gene regulatory networks based on the DEMs and the two condition-specific

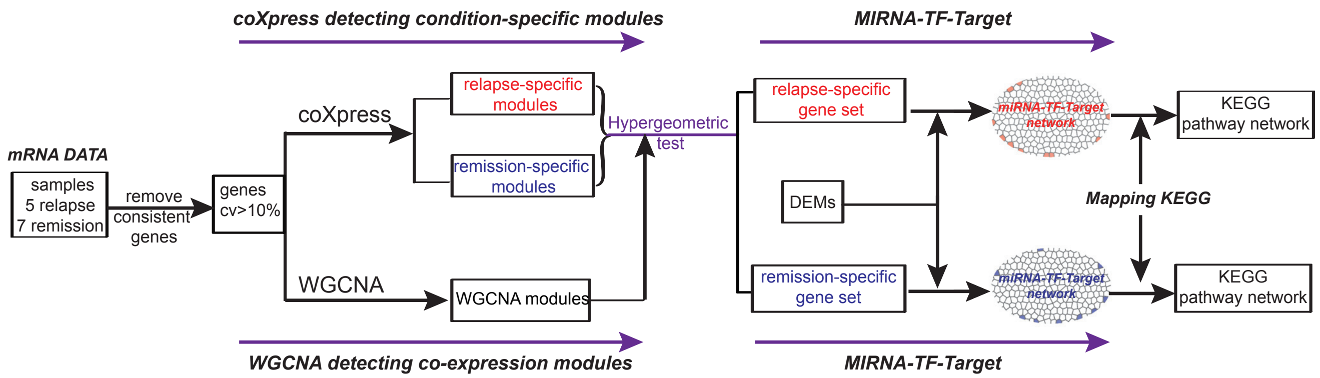


**Figure 3. The Workflow for Co-expression and miRNA-TF-Gene Network Analyses**

gene sets, respectively. A total of 611 nodes (58 miRNAs, 52 TFs, and 501 genes) with 5,371 pairs were found in the remission-specific network (Figure S1A), while the relapse-specific regulatory network contained 13,920 regulating pairs consisting of 58 miRNAs, 93 TFs, and 874 genes (Figure S1B). Furthermore, to detect key regulators and genes within relapse and remission conditions, we performed pathway crosstalk analysis based on the above networks and rebuilt core regulatory sub-networks (Figures 5A, 5B, and 6A).

Totally, the relapse sub-network consisted of 57 miRNAs, 74 genes, and 11 TFs (Figure S2), and most genes were related to cancer, immunity, and apoptosis and signal transduction pathways (Figure 5A). Furthermore, genes that acted as hub nodes connecting multiple biological processes and pathways in the relapse sub-network may contribute to the relapse of pediatric T-ALL. For example, *NOTCH2* reported as an oncogene promoting leukemia transformation,[29] here cross-linked the NOTCH signaling, miRNAs in cancer, and Th1/Th2 cell differentiation pathways in the relapse sub-network (Figure 5A). miR-1246/1248/22-5p combined with TFs *WT1*/*SOX4*/*REL* regulating 578 genes acted as core modules in the relapse-specific network (Figure S2), and 65 of these genes were DEGs in the relapse versus remission group comparison. Furthermore, 24 of 65 DEGs were stably expressed between relapse and remission groups, which may imply they were closely relevant to the relapse of T-ALL. For example, *NOTCH2* was reported to promote cell proliferation in T-ALL cell lines;[30] the stably upregulated *REL* as a TF could decrease cell apoptosis of hematologic malignancies.[31] Meanwhile, TF *WT1*, positively regulating the expression of *CD95L* to stimulate CD95L-mediated cell death,[32] was the hub node with most connections in our relapse-specific network (Figure S2).

On the other hand, the remission sub-network consisted of 55 miRNAs, 67 genes, and 11 TFs (Figure 5B), while 8 genes were stably differentially expressed (Table S1). Notably, 18 genes acted as cross-talk hubs connecting several crucial pathways in the development of T-ALL (Figure 6A). For example, *CTBP2*, as the most significantly DEG downregulated in the relapse group, cross-linked NOTCH- and Wnt-signaling pathways in the remission sub-network (Figure 6A), which were closely associated with the development of T-ALL.[33] Meanwhile, *CTBP2* was a co-repressor for the NOTCH-signaling pathway,[34] and it was targeted by miR-1246/1248 upregulated in the relapse group (Figures 2A and 5B). Meanwhile, TF *MYCN* is a central regulator of multiple vital cellular processes, and it has been described as an oncogene in multiple cancer types.[35] In our remission sub-network, *MYCN* was predictively targeted by tumor suppressor miR-429 (Figure 5B) and significantly downregulated in the remission group. Furthermore, *MFHAS1*, reported to promote the progress of cancer,[36] was regulated by *MYCN*. Interestingly, both *MYCN* and *MFHAS1* may be targets of miR-429, which implied miR-429-*MYCN*-*MFHAS1* may form an FFL taking part in the progress of T-ALL (Figure 5B).

## miR-1246/1248 Could Promote Cell Proliferation in the T-ALL Cell Line through the NOTCH2 Pathway

To evaluate the biological effects of miR-1246/1248 underlying the relapse of pediatric T-ALL, we performed gain-of-function and loss-of-function experiments using miRNA mimics and inhibitors on the Jurkat cell line. First, Jurkat cells were infected with lentiviral expression vectors to overexpress miR-1246 and miR-1248, respectively, and the cell growth was evaluated at 24 hr after infection. A significant increase of cell proliferation rates was observed after transfecting miR-1246 or miR-1248 mimics into the Jurkat cells compared with the control group (Figures 6B and 6C). Next, we transfected the Jurkat cells with miR-1246 and miR-1248 inhibitors, respectively. As a result, we found the anti-miR-1246 and anti-miR-1248 had opposite effects on the Jurkat cell line, which significantly decreased cell proliferation (Figures 6B and 6C). Combining the above results, we inferred that the miR-1246/1248 could promote cell proliferation in the T-ALL cell line, which may result in the relapse of T-ALL.

To further investigate the mechanism through which miR-1246 and miR-1248 promote cell proliferation, we focused on the crucial gene *NOTCH2* in the relapse sub-network, and we determined the effects of *NOTCH2* on Jurkat cells. Jurkat cells were transfected with small interfering RNAs (siRNAs) of *NOTCH2* and miR-1246/miR-1248

**Table 1. The Significant Modules Detected by coXpress under Relapse and Remission Conditions**

| Group | N | pr.g1 | pr.g2 | Mean.cor1 | Mean.cor2 |
|---|---|---|---|---|---|
| coXpress Modules Related to Relapse | | | | | |
| 7 | 241 | 0 | 0.06 | 0.825 | 0.048 |
| 2 | 137 | 0 | 0.71 | 0.750 | 0.027 |
| 13 | 113 | 0 | 0.89 | 0.727 | 0.012 |
| 6 | 112 | 0 | 0.26 | 0.801 | 0.05 |
| 3 | 56 | 0 | 0.45 | 0.749 | 0.029 |
| 5 | 51 | 0 | 0.21 | 0.756 | 0.046 |
| 21 | 32 | 0 | 0.19 | 0.738 | 0.050 |
| coXpress Modules Related to Remission | | | | | |
| 5 | 82 | 0.95 | 0 | 0.002 | 0.813 |
| 10 | 58 | 0.34 | 0 | 0.032 | 0.694 |
| 39 | 47 | 0.79 | 0 | 0.007 | 0.724 |
| 18 | 45 | 0.09 | 0 | 0.072 | 0.848 |
| 7 | 41 | 0.06 | 0 | 0.101 | 0.822 |
| 34 | 35 | 0.53 | 0 | 0.020 | 0.828 |
| 6 | 34 | 0.59 | 0 | 0.020 | 0.700 |
| 36 | 32 | 0.06 | 0 | 0.106 | 0.771 |

Group is the group number and N is the group size (gene number). pr.g1 and pr.g2 are the probability of randomness statistics for the relapse and remission subsets, respectively. The mean.corr1 and mean.corr2 are the mean pairwise correlation coefficients for the genes in relapse and remission, respectively. The table has been screened by pr.g1 $\leq$ 0.05, pr.g2 $\geq$ 0.05, and N $\geq$ 30.

mimic, and then we calculated the cell proliferation rate. As expected, the expression of *NOTCH2* was repressed by its siRNAs (Figure S3A), and Jurkat cells transfected with NOTCH2_siRNA showed a significant retardation of cell growth (Figure 6B). However, even if the miR-1246 or miR-1248 was overexpressed, the proliferations of Jurkat cells were nearly unchanged with the knockdown of *NOTCH2* (Figures 6B and 6C). These results implied the oncogenic roles of miR-1246/1248 may rely on the signal transduction of *NOTCH2*, and *NOTCH2* cooperating with miR-1246/1248-promoting cell proliferation may contribute to the relapse of T-ALL.

### Potential Drug-Targeting miRNAs Related to the Relapse of T-ALL

Previous study demonstrated that miRNAs as important transcriptional regulators mediate the progression of T-ALL and may serve as potential therapeutic targets.[37] We investigated whether BFM therapy could have effects on miRNAs through drug-miRNA interactions. Notably, we found that no DEMs were targeted by BFM drugs in the SM2miR database.[38] However, some other drugs could target DEMs and may serve as useful supplements for classic therapy, such as arsenic trioxide. Arsenic trioxide was reported inhibiting the progress of leukemia by upregulating the expression of some miRNAs, including miR-150-5p,[39] which was among downregulated DEMs in the relapse group (Figure 7). Furthermore, we constructed an miRNA-drug network to reveal the potential drug targets (Figure 7), which provided the possible clues for molecular therapy for pediatric T-ALL. For instance, the upregulated miR-1246 was targeted by ascorbate, trastuzumab, and 5-fluorouracil (Figure 7), and ascorbate regulated leukaemogenesis,[40] while trastuzumab was engaged in very high-risk-relapsed adult B cell-ALL (B-ALL).[41] The 5-fluorouracil can significantly dysregulate the expression level of miR-1246 in cancer cells.[42] Moreover, the miR-1246/1248-*NOTCH2* may provide a clue for the relapse of pediatric T-ALL, and 5-fluorouracil/ascorbate/trastuzumab targeting miR-1246 may serve as potential candidates for the T-ALL.

## DISCUSSION

The diagnosis and treatment for the relapse of pediatric T-ALL remain a challenge because of its heterogeneity and malignancy. In current study, we investigated potential molecular mechanisms for the relapse of pediatric T-ALL by integrating co-expression and miRNA-TF-gene network analysis. We detected several key genes and regulators, such as miR-1246, miR-1248, and miR-429 and *NOTCH2*/*MYCN*/*MFHAS1*, may play vital roles for T-ALL relapse. Moreover, we validated miR-1246/1248-*NOTCH2* could cooperate to promote cell proliferation in the Jurkat cell line, which may contribute to the relapse of pediatric T-ALL. The miRNA-drug network analysis could provide preliminary clues for precision medicine approaches on pediatric T-ALL.

Previous research mainly focused on the paired samples (diagnosis and relapse after treatment) to explore the mechanism underlying the relapse of pediatric T-ALL. However, for the heterogeneity of T-ALL, studies based on initially diagnosed samples with known follow-up relapse/remission after therapy may provide new insights for the precise treatment of pediatric T-ALL. Meanwhile, dissection of the differential co-expression pattern between specific conditions could provide additional information, which cannot be detected by standard co-expression methods.[43] Our study based on pre-treatment samples (leukemia state) has discovered specific regulatory networks relevant to the relapse/remission of pediatric T-ALL. Genes emerged in relapse or remission network have meant to be more closely associated with the specific biological processes. MiR-1246/1248 combined with *NOTCH2* and TFs *WT1*/*SOX4*/*REL* acted as core modules in the relapse-specific network. *NOTCH2* as a crucial crosstalk gene connected multiple pathways, including the miRNAs in cancer and the NOTCH-signaling pathway (Figure 5A), and it was an important carcinogenic pathway in leukemia.[29]

Cell experiments validated that miR-1246/1248 could promote Jurkat cell proliferation by NOTCH2 signal. The upregulation of miR-1246 and -1248 may suppress the expression of CTBP2, which is a co-repressor of the NOTCH2 signal pathway. Hence, the miR-1246/1248 may activate the downstream signaling pathway of NOTCH through suppressing the expression of *CTBP2*. Meanwhile, TFs *WT1*/*SOX4*/*REL* may regulate *NOTCH2* takes part in the process of T-ALL. These results implied that the miR-1246/1248-*NOTCH2* may contribute to the relapse of T-ALL and the relapse-specific network could play vital roles in T-ALL. On the other hand, FFL

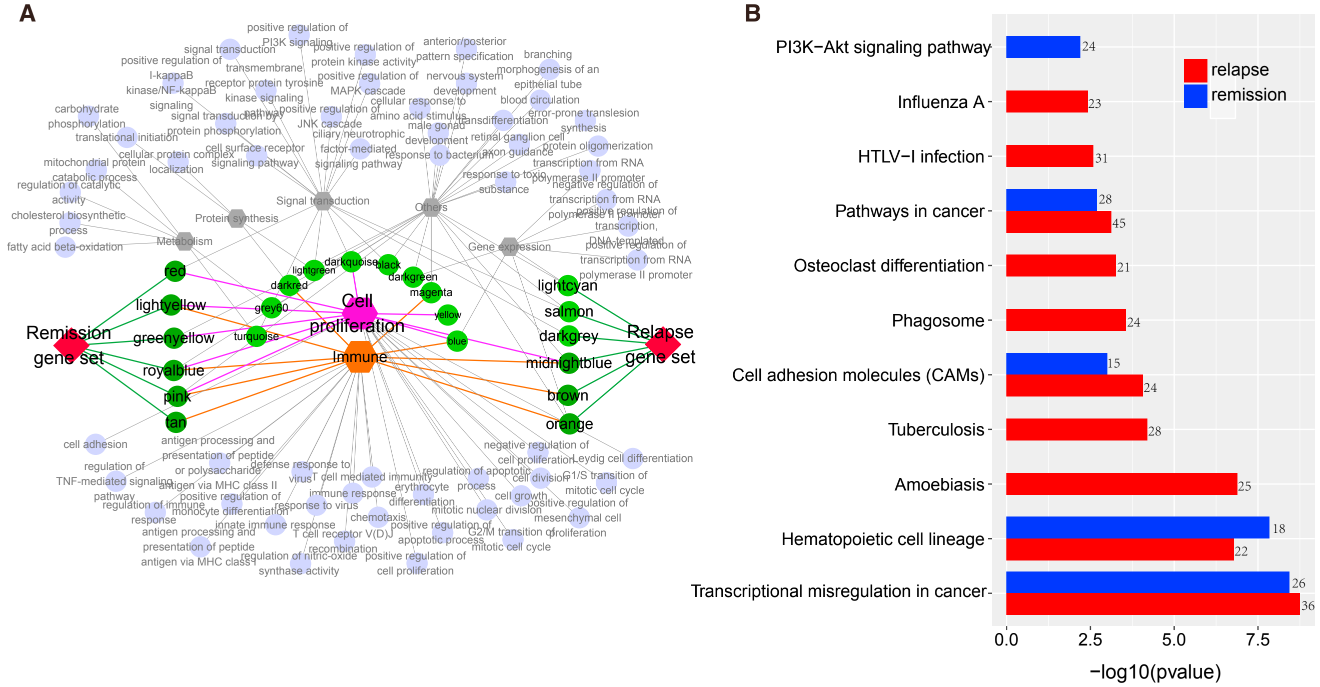


**Figure 4. The Function Analysis of Modules**

(A) The GO enrichment of 22 WGCNA modules (p value < 0.05). Purple, GO terms; green and dark green, modules; gray, function type. (B) KEGG pathways enriched by the specific gene sets (relapse/remission gene sets) (p value < 0.01).

miR-429-*MYCN*-*MFHAS1* detected in the remission sub-network may play vital roles in the process of T-ALL. MiR-429 was significantly upregulated in the remission group, which may result in the downregulation of its target *MFHAS1* (Figure 5B). *MFHAS1*, regulated by TF *MYCN* in our remission sub-network, may be a significant prognosis factor for AML in The Cancer Genome Atlas (TCGA) cohort (Figure 3B) and could promote the progress of cancer.[36] Other key nodes in our regulatory networks were also closely related to the process of T-ALL. For example, *DUSP6* as a phosphatase, regulated phosphorylation of its downstream gene *ERK1/2* to promote cell proliferation,[44] and it may contribute to the relapse by crosstalk with *WT1* through transcriptional misregulation in cancer pathway (Figure 5A). Moreover, genes involved in the pathway crosstalk may play core roles in the relapse of T-ALL, such as *BCL2*, which was a potential therapeutic strategy for T-ALL (Figure 5A).[45]

Furthermore, studies on disease mechanisms will be eventually fed back to effective drug designs and therapeutic strategies. Our miRNA-drug network provided some potential drugs for the relapsed T-ALL. For example, the upregulated miR-1246 was targeted by three drugs (5-fluorouracil, ascorbate, and trastuzumab) (Figure 7). The ascorbate was reported to regulate the leukemogenesis,[40] and thus the ascorbate may regulate the expression level of miR-1246 in relapse, leading to the relief of T-ALL. The trastuzumab was reported to allow for some responses in very high-risk-relapsed adult B-ALL patients. Moreover, in the miRNA-drug network, we found arsenic trioxide, as an effective anti-leukemia drug, promoted the upregulation of miR-150-5p and miR-96-5p (Figure 7),[39] which were downregulated in relapsed samples. Thus, arsenic trioxide may relieve the relapse of T-ALL patients by upregulating the miR-150-5p and miR-96-5p.[39] Similar to these observations, our drug target network may provide some potential drug targets and drugs for the treatment of T-ALL.

In summary, in this study, we integrated differential co-expression and miRNA-TF-gene regulatory network analysis to reveal the possible mechanisms for the relapse of T-ALL. Our finding indicated that the upregulated miR-1246/1248 may cooperate with *NOTCH2* to promote the relapse of T-ALL by the *NOTCH2*-signaling pathway, and they provided some miRNA-drug pair information, which may be helpful for the precise treatment of T-ALL. Based on the differential transcriptome profiling between the relapse and remission specimens, we suggested that different treatment protocols should be applied to patients to achieve precise treatments.

## MATERIALS AND METHODS

### Data Collection, Pre-processing, and Differential Expression Analysis

Gene expression profiles of pediatric pre-treatment T-ALL cases (relapse, n = 5; remission, n = 7) were obtained from the European Bioinformatics Institute (EBI): E-TABM-255, while miRNA data (relapse, n = 5; remission, n = 7) were from GEO: GSE45839. Public

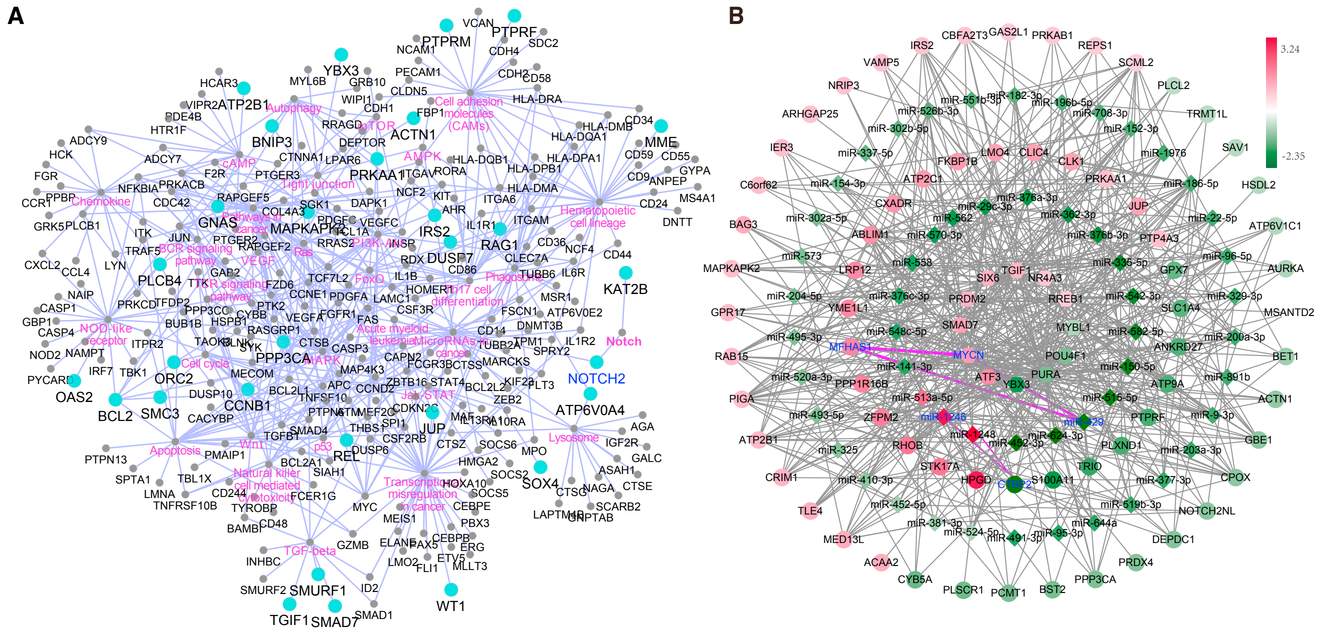


**Figure 5. The Regulatory Network and Crosstalk Analysis for Gene Set**

(A) KEGG pathway network formed by relapse gene sets. Blue nodes, DEGs; pink labels, KEGG pathways. (B) miRNA-TF-gene regulatory network for DEGs in remission gene sets. Hexagon, miRNA; diamond, transcriptional factor; ellipse, gene.

pediatric pre-treatment T-ALL cases were used in this study (range of 2–13.4 years). All patients were accepted for BFM therapy protocol. BFM protocol included the following: 4 standard drugs (cyclophosphamide, 6-mercaptopurine, cytarabine, and methotrexate) and 6 improved BFM drugs (L-asparaginase, dexamethasone, doxorubicin, prednisone, 6-thioguanine, and vincristine).[46] Both gene and miRNA data were normalized using the robust multi-array average (RMA) method. Genes were annotated according to information, and multiple probes for a single gene were treated by k.total,[47] while miRNAs were identified with miRXplore_v4.0 and miRBase (v21, GRCh38). DEGs and DEMs were detected using limma package with the default parameters and the following criteria: |fold change (FC)| > 1.5 and adjusted p value < 0.05.[48]

### Gene Module-Based Analysis of the Relapse and Remission Samples

Gene co-expression modules were identified by the WGCNA package. First, we filtered out genes with low CV less than 10% in relapse and remission specimens. Next, the remaining genes were used to construct weighted gene correlation network. Here, soft-thresholding power β of co-expression network was chosen by the criterion of scale-free topology with $R^2$ cutoff (0.9), and each branch in the dendrogram represents a module in the network. To obtain modules with proper biological functions, the parameters (minModuleSize = 20; minimum height = 0.2) were used to cut the tree.

To identify groups of genes that displayed differential co-expression patterns, which distinguished between the relapse and remission samples, we also applied coXpress on the gene expression dataset (parameter: s = pearson, m = average, h = 0.4). The conditionally related modules (relapse/remission) were identified for genes screened using the coXpress package, which could detect differential co-expression gene modules only highly associated with a given condition but little or no relation in the other.[17] Those significantly differential co-expression gene modules in relapse samples were selected for further study (with N > 30, pr.g1 < 0.05, pr.g2 > 0.05). To find the opposite condition, the procedure was repeated but based on remission datasets.[17]

Finally, to obtain comprehensive gene modules that contribute to the relapse of T-ALL, we combined coXpress with WGCNA modules together by measuring the significant overlapping based on the hypergeometric probability. For example, if one differential module is composed of *k* genes, and *l* genes are detected in one of the WGCNA modules, the probability is obtained by hypergeometric test formula, where *M* and *N* represent the total number of genes in the corresponding module, respectively. Here, we set the significance level at 0.01.

$$P\ (X \le k) = 1 - \sum_{i=0}^{k} \frac{\binom{M}{i}\binom{N-M}{k-i}}{\binom{N}{k}}$$

### Regulatory Network Analysis for the Significant Modules

We constructed a comprehensive regulatory network based on DEGs (within the significant differential modules) and DEMs by the method

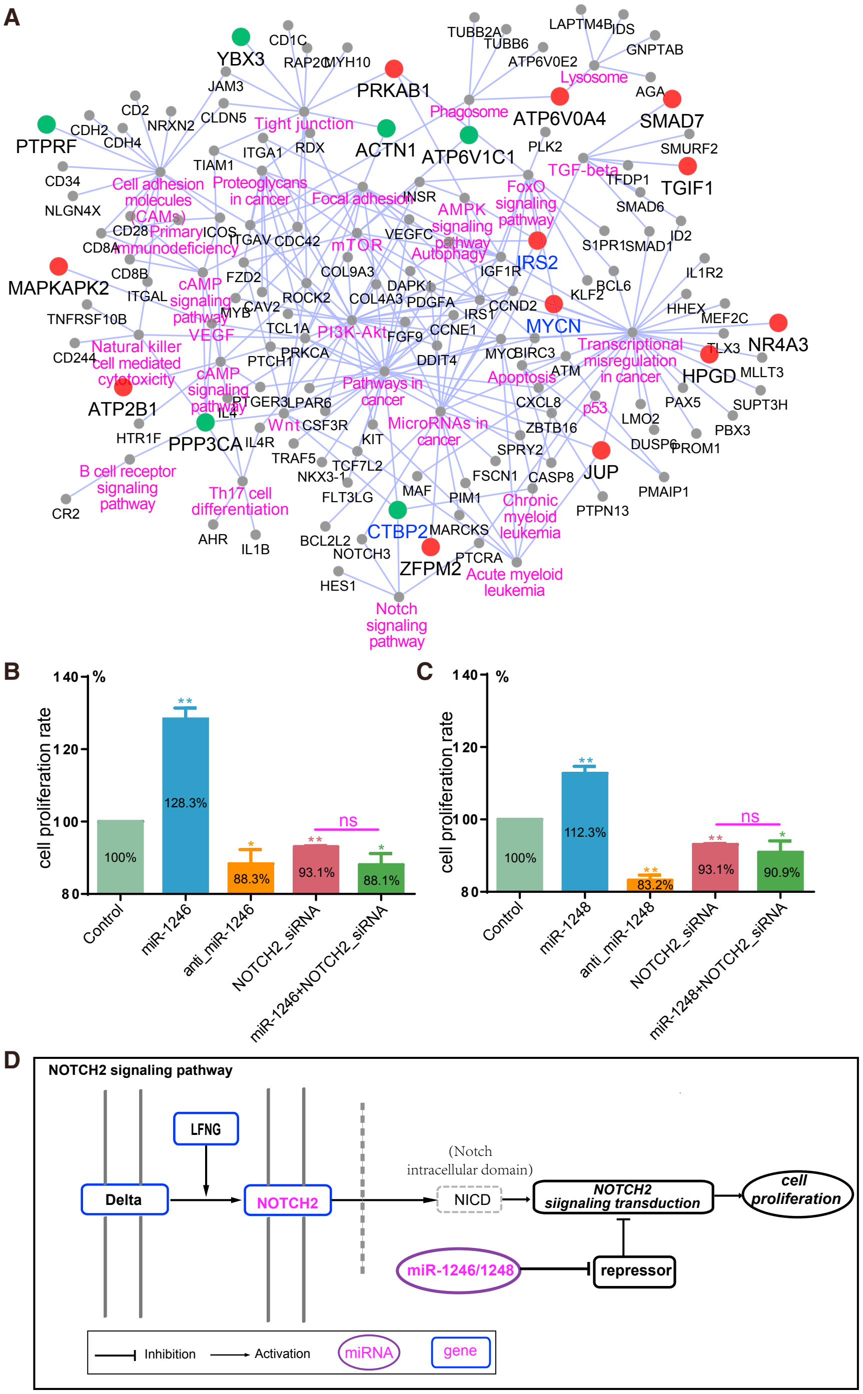


**Figure 6. The Crosstalk Analysis for Remission Gene Set and Experimental Verification**

(A) KEGG pathway network formed by remission gene sets. Red nodes, upregulation in relapse; green nodes, downregulation in relapse; gray nodes, non-DEGs; pink labels, KEGG pathways. (B and C) Effects of miR-1246 (B) and miR-1248 (C) and NOTCH2 on cell proliferation in the Jurkat cell line. The numbers in the bar represent the means. Values represent the means ± SD (**p < 0.001 and *p < 0.01; ns, not significant). (D) NOTCH2-signaling pathway and possible mechanism of miR-1246/1248 on T-ALL relapse.

described in our previous studies.[49] The miRNA-target information was obtained from the miRwalk2.0,[50] while the TF information was from the AnimalTFDB.[51,52]

## Statistical Analysis, Functional Enrichment, and Visualization

Gene ontology (GO) and KEGG pathway enrichment analyses were performed on DAVID (https://david.ncifcrf.gov/).[53] For DEMs, functional enrichment analysis was conducted by the DIANA-miRPath version (v.)3.0.[54] All networks were visualized by Cytoscape (version 3.4.0) and ClueGO plugin.[55] NetworkAnalyzer was used to calculate the degrees of network for identifying the HUB factors.[56] The disease-free survival (DFS) analysis was performed on the AML dataset (TCGA) using survival package (p value < 0.1, log rank test). The interactions of miRNAs and small molecules were obtained from the SM2miR database.[38]

## Cell Culture and Transfection of siRNA and Mimics

The Jurkat human T-ALL cell line was obtained from the China Center for Type Culture Collection (CCTCC, Wuhan, China). Cells were cultured in a complete medium (RPMI-1640 supplement with 10% fetal bovine serum [FBS] and 1% penicillin/streptomycin) in a 5% $CO_2$ 37°C incubator. Approximately 5 × $10^5$ cells were seeded in six-well plates with complete RPMI-1640 medium. Three different pre-designed siRNAs (Ribo siRNA) targeting NOTCH2 (siNOTCH2: stB0007281A, stB0007281B, and stB0007281C) were purchased from RIBOBIO (Guangzhou, China; details in Table S3). Each siRNA or miRNA mimics, negative control (NC), and reagent (RIBOBIO, Guangzhou, China) were transfected at a concentration of 50 nM according to the manufacturer's instructions. After transfection, the cells were quickly transferred into culture medium. Cells were harvested after 24 hr to extract total RNA for RT-PCR.

## Real-Time qRT-PCR

Total RNA was extracted with TRIzol (Invitrogen) to synthesize cDNA using PrimeScript RT Reagent Kit (Takara). Real-time RT-PCR was performed on StepOne Real-Time PCR System (Life

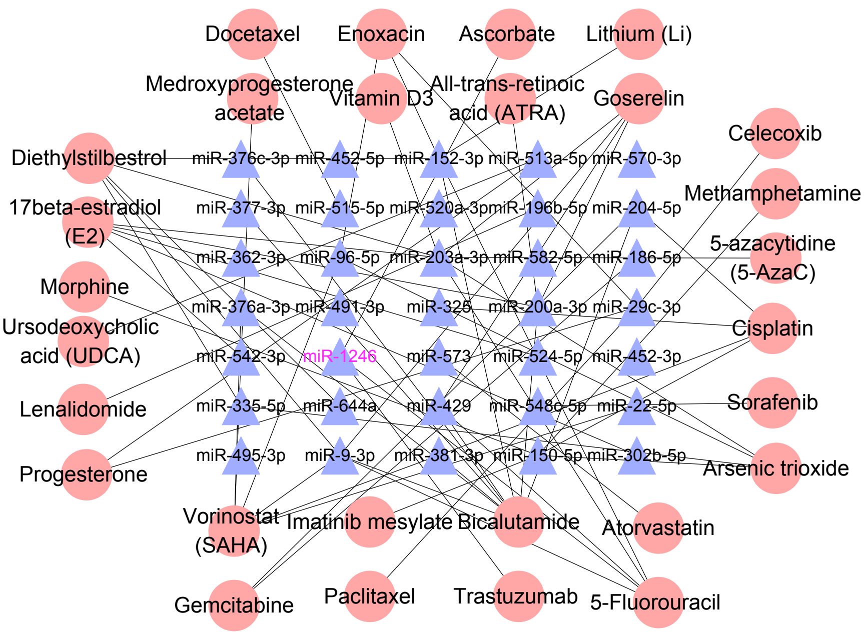


**Figure 7. Drug Target Analysis**

Drug-miRNA network was constructed by drugs approved by the US Food and Drug Administration in the SM2miR database and DEMs.

Technologies). The final reaction volume (10 μL) included 1 μL PrieScript RT Enzyme MixI (Takara), 1 μL primers (forward and reverse), 4 μL 5*PrimrScript Buffer 2, and 4 μL RNase-free $H_2O$. All experiments were repeated at least three times. Relative expression was analyzed using the ΔΔCt method. The primer sequences used for RT-PCR are shown in Table S4 (Genecreate, Wuhan, China).

### Cell Proliferation Assay

The effects of transfections on short-term growth were examined using a colorimetric WST-8 assay (Cell Counting Kit-8, Beyotime Biotechnology, Shanghai, China). Cells (2–4 × $10^4$ cells/well) were cultured in 0.1 mL 10% FBS-supplemented RPMI-1640 medium in 96-well culture plates. The NC, miRNA mimics, and siRNAs were transfected into cells after 24 hr, and 10 μL CCK-8 was added to the cells another day. The optical density (OD) was then measured using an ELISA plate reader (Diatek) to determine the cell number. The cell growth is shown as a percentage of the mean OD value compared with the control. Student's t test was used to determine the statistical significance.

## SUPPLEMENTAL INFORMATION

Supplemental Information includes three figures and four tables and can be found with this article online at https://doi.org/10.1016/j.omtn.2018.05.003.

## AUTHOR CONTRIBUTIONS

Conceptualization, A.-Y.G. and Q.Z.; Methodology, A.-Y.G. and Q.Z.; Formal Analysis, Q.Z., M.L., M.X., F.H., Z.M., and Z.C.; Writing – Original Draft, M.L.; Writing – Review & Editing, A.-Y.G. and Q.Z.; Funding Acquisition, A.-Y.G.; Supervision, A.-Y.G. and Q.Z.

## ACKNOWLEDGMENTS

We acknowledge the research funding from the National Key Research and Development Program of China (2017YFA0700403) and the National Natural Science Foundation of China (NSFC) (31471247 and 31771458).

## REFERENCES

1. Pui, C.-H., Robison, L.L., and Look, A.T. (2008). Acute lymphoblastic leukaemia. Lancet *371*, 1030–1043.
2. Aifantis, I., Raetz, E., and Buonamici, S. (2008). Molecular pathogenesis of T-cell leukaemia and lymphoma. Nat. Rev. Immunol. *8*, 380–390.
3. Seibel, N.L., Steinherz, P.G., Sather, H.N., Nachman, J.B., Delaat, C., Ettinger, L.J., Freyer, D.R., Mattano, L.A., Jr., Hastings, C.A., Rubin, C.M., et al. (2008). Early post-induction intensification therapy improves survival for children and adolescents with high-risk acute lymphoblastic leukemia: a report from the Children's Oncology Group. Blood *111*, 2548–2555.
4. Durinck, K., Goossens, S., Peirs, S., Wallaert, A., Van Loocke, W., Matthijssens, F., Pieters, T., Milani, G., Lammens, T., Rondou, P., et al. (2015). Novel biological insights in T-cell acute lymphoblastic leukemia. Exp. Hematol. *43*, 625–639.
5. Weng, A.P., Ferrando, A.A., Lee, W., Morris, J.P., 4th, Silverman, L.B., Sanchez-Irizarry, C., Blacklow, S.C., Look, A.T., and Aster, J.C. (2004). Activating mutations of *NOTCH1* in human T cell acute lymphoblastic leukemia. Science *306*, 269–271.
6. Hogan, L.E., Meyer, J.A., Yang, J., Wang, J., Wong, N., Yang, W., Condos, G., Hunger, S.P., Raetz, E., Saffery, R., et al. (2011). Integrated genomic analysis of relapsed childhood acute lymphoblastic leukemia reveals therapeutic strategies. Blood *118*, 5218–5226.
7. Gottardo, N.G., Hoffmann, K., Beesley, A.H., Freitas, J.R., Firth, M.J., Perera, K.U., de Klerk, N.H., Baker, D.L., and Kees, U.R. (2007). Identification of novel molecular prognostic markers for paediatric T-cell acute lymphoblastic leukaemia. Br. J. Haematol. *137*, 319–328.
8. Martelli, A.M., Lonetti, A., Buontempo, F., Ricci, F., Tazzari, P.L., Evangelisti, C., Bressanin, D., Cappellini, A., Orsini, E., and Chiarini, F. (2014). Targeting signaling pathways in T-cell acute lymphoblastic leukemia initiating cells. Adv. Biol. Regul. *56*, 6–21.

9. Gianfelici, V., Chiaretti, S., Demeyer, S., Di Giacomo, F., Messina, M., La Starza, R., Peragine, N., Paoloni, F., Geerdens, E., Pierini, V., et al. (2016). RNA sequencing unravels the genetics of refractory/relapsed T-cell acute lymphoblastic leukemia. Prognostic and therapeutic implications. Haematologica *101*, 941–950.

10. Cui, T., Zhang, L., Huang, Y., Yi, Y., Tan, P., Zhao, Y., Hu, Y., Xu, L., Li, E., and Wang, D. (2018). MNDR v2.0: an updated resource of ncRNA-disease associations in mammals. Nucleic Acids Res. *46* (D1), D371–D374.

11. Wu, D., Huang, Y., Kang, J., Li, K., Bi, X., Zhang, T., Jin, N., Hu, Y., Tan, P., Zhang, L., et al. (2015). ncRDeathDB: A comprehensive bioinformatics resource for deciphering network organization of the ncRNA-mediated cell death system. Autophagy *11*, 1917–1926.

12. Fragoso, R., Mao, T., Wang, S., Schaffert, S., Gong, X., Yue, S., Luong, R., Min, H., Yashiro-Ohtani, Y., Davis, M., et al. (2012). Modulating the Strength and Threshold of NOTCH Oncogenic Signals by *mir-181a-1/b-1*. PLoS Genet. *8*, e1002855.

13. Avigad, S., Verly, I.R.N., Lebel, A., Kordi, O., Shichrur, K., Ohali, A., Hameiri-Grossman, M., Kaspers, G.J., Cloos, J., Fronkova, E., et al. (2016). miR expression profiling at diagnosis predicts relapse in pediatric precursor B-cell acute lymphoblastic leukemia. Genes Chromosomes Cancer *55*, 328–339.

14. Shen, Y., Park, C.S., Suppipat, K., Mistretta, T.-A., Puppi, M., Horton, T.M., Rabin, K., Gray, N.S., Meijerink, J.P.P., and Lacorazza, H.D. (2017). Inactivation of KLF4 promotes T-cell acute lymphoblastic leukemia and activates the MAP2K7 pathway. Leukemia *31*, 1314–1324.

15. Liau, W.S., Tan, S.H., Ngoc, P.C.T., Wang, C.Q., Tergaonkar, V., Feng, H., Gong, Z., Osato, M., Look, A.T., and Sanda, T. (2017). Aberrant activation of the GIMAP enhancer by oncogenic transcription factors in T-cell acute lymphoblastic leukemia. Leukemia *31*, 1798–1807.

16. Langfelder, P., and Horvath, S. (2008). WGCNA: an R package for weighted correlation network analysis. BMC Bioinformatics *9*, 559.

17. Watson, M. (2006). CoXpress: differential co-expression in gene expression data. BMC Bioinformatics *7*, 509.

18. Lin, Y., Zhang, Q., Zhang, H.-M., Liu, W., Liu, C.-J., Li, Q., and Guo, A.Y. (2015). Transcription factor and miRNA co-regulatory network reveals shared and specific regulators in the development of B cell and T cell. Sci. Rep. *5*, 15215.

19. Ye, H., Liu, X., Lv, M., Wu, Y., Kuang, S., Gong, J., Yuan, P., Zhong, Z., Li, Q., Jia, H., et al. (2012). MicroRNA and transcription factor co-regulatory network analysis reveals miR-19 inhibits CYLD in T-cell acute lymphoblastic leukemia. Nucleic Acids Res. *40*, 5201–5214.

20. Knight, T., and Irving, J.A.E. (2014). Ras/Raf/MEK/ERK Pathway Activation in Childhood Acute Lymphoblastic Leukemia and Its Therapeutic Targeting. Front. Oncol. *4*, 160.

21. Kong, G., Du, J., Liu, Y., Meline, B., Chang, Y.-I., Ranheim, E.A., Wang, J., and Zhang, J. (2013). Notch1 gene mutations target KRAS G12D-expressing CD8+ cells and contribute to their leukemogenic transformation. J. Biol. Chem. *288*, 18219–18227.

22. Yuan, T., Yang, Y., Chen, J., Li, W., Li, W., Zhang, Q., Mi, Y., Goswami, R.S., You, J.Q., Lin, D., et al. (2017). Regulation of PI3K signaling in T-cell acute lymphoblastic leukemia: a novel PTEN/Ikaros/miR-26b mechanism reveals a critical targetable role for PIK3CD. Leukemia *31*, 2355–2364.

23. Classen, C.F., Ushmorov, A., Bird, P., and Debatin, K.M. (2004). The granzyme B inhibitor PI-9 is differentially expressed in all main subtypes of pediatric acute lymphoblastic leukemias. Haematologica *89*, 1314–1321.

24. Kanehisa, M., and Goto, S. (2000). KEGG: kyoto encyclopedia of genes and genomes. Nucleic Acids Res. *28*, 27–30.

25. Girardi, T., Vicente, C., Cools, J., and De Keersmaecker, K. (2017). The genetics and molecular biology of T-ALL. Blood *129*, 1113–1123.

26. Stumpel, D.J.P.M., Schotte, D., Lange-Turenhout, E.A., Schneider, P., Seslija, L., de Menezes, R.X., Marquez, V.E., Pieters, R., den Boer, M.L., and Stam, R.W. (2011). Hypermethylation of specific microRNA genes in MLL-rearranged infant acute lymphoblastic leukemia: major matters at a micro scale. Leukemia *25*, 429–439.

27. Liang, H., Zheng, Q.-L., Fang, P., Zhang, J., Zhang, T., Liu, W., Guo, M., Robinson, C.L., Chen, S.B., Chen, X.P., et al. (2017). Targeting the PI3K/AKT pathway via GLI1 inhibition enhanced the drug sensitivity of acute myeloid leukemia cells. Sci. Rep. *7*, 40361.

28. Waning, D.L., Mohammad, K.S., and Guise, T.A. (2013). Cancer-associated osteoclast differentiation takes a good look in the miR(NA)ror. Cancer Cell *24*, 407–409.

29. Wickremasinghe, R.G., Prentice, A.G., and Steele, A.J. (2011). p53 and Notch signaling in chronic lymphocytic leukemia: clues to identifying novel therapeutic strategies. Leukemia *25*, 1400–1407.

30. Okuhashi, Y., Itoh, M., Nara, N., and Tohda, S. (2013). NOTCH knockdown affects the proliferation and mTOR signaling of leukemia cells. Anticancer Res. *33*, 4293–4298.

31. Turco, M.C., Romano, M.F., Petrella, A., Bisogni, R., Tassone, P., and Venuta, S. (2004). NF-kappaB/Rel-mediated regulation of apoptosis in hematologic malignancies and normal hematopoietic progenitors. Leukemia *18*, 11–17.

32. Bourkoula, K., Englert, C., Giaisi, M., Köhler, R., Krammer, P.H., and Li-Weber, M. (2014). The Wilms' tumor suppressor WT1 enhances CD95L expression and promotes activation-induced cell death in leukemic T cells. Int. J. Cancer *134*, 291–300.

33. Staal, F.J.T., van Dongen, J.J.M., and Langerak, A.W. (2007). Novel insights into the development of T-cell acute lymphoblastic leukemia. Curr. Hematol. Malig. Rep. *2*, 176–182.

34. Oswald, F., Winkler, M., Cao, Y., Astrahantseff, K., Bourteele, S., Knöchel, W., and Borggrefe, T. (2005). RBP-Jkappa/SHARP recruits CtIP/CtBP corepressors to silence Notch target genes. Mol. Cell. Biol. *25*, 10379–10390.

35. Beltran, H. (2014). The N-myc Oncogene: Maximizing its Targets, Regulation, and Therapeutic Potential. Mol. Cancer Res. *12*, 815–822.

36. Chen, W., Xu, Y., Zhong, J., Wang, H., Weng, M., Cheng, Q., Wu, Q., Sun, Z., Jiang, H., Zhu, M., et al. (2016). MFHAS1 promotes colorectal cancer progress by regulating polarization of tumor-associated macrophages via STAT6 signaling pathway. Oncotarget *7*, 78726–78735.

37. Kumar, V., Palermo, R., Talora, C., Campese, A.F., Checquolo, S., Bellavia, D., Tottone, L., Testa, G., Miele, E., Indraccolo, S., et al. (2014). Notch and NF-kB signaling pathways regulate miR-223/FBXW7 axis in T-cell acute lymphoblastic leukemia. Leukemia *28*, 2324–2335.

38. Liu, X., Wang, S., Meng, F., Wang, J., Zhang, Y., Dai, E., Yu, X., Li, X., and Jiang, W. (2013). SM2miR: a database of the experimentally validated small molecules' effects on microRNA expression. Bioinformatics *29*, 409–411.

39. Ghaffari, S.H., Bashash, D., Dizaji, M.Z., Ghavamzadeh, A., and Alimoghaddam, K. (2012). Alteration in miRNA gene expression pattern in acute promyelocytic leukemia cell induced by arsenic trioxide: a possible mechanism to explain arsenic multi-target action. Tumour Biol. *33*, 157–172.

40. Agathocleous, M., Meacham, C.E., Burgess, R.J., Piskounova, E., Zhao, Z., Crane, G.M., Cowin, B.L., Bruner, E., Murphy, M.M., Chen, W., et al. (2017). Ascorbate regulates haematopoietic stem cell function and leukaemogenesis. Nature *549*, 476–481.

41. Chevallier, P., Robillard, N., Charbonnier, A., Raffoux, E., Maury, S., Carras, S., Chabrot, C., Fohrer, C., Bernard, M., Blade, J.S., et al. (2012). Trastuzumab for treatment of refractory/relapsed HER2-positive adult B-ALL: results of a phase 2 GRAALL study. Blood *119*, 2474–2477.

42. Shah, M.Y., Pan, X., Fix, L.N., Farwell, M.A., and Zhang, B. (2011). 5-Fluorouracil drug alters the microRNA expression profiles in MCF-7 breast cancer cells. J. Cell. Physiol. *226*, 1868–1878.

43. de la Fuente, A. (2010). From 'differential expression' to 'differential networking' - identification of dysfunctional regulatory networks in diseases. Trends Genet. *26*, 326–333.

44. Maillet, M., Purcell, N.H., Sargent, M.A., York, A.J., Bueno, O.F., and Molkentin, J.D. (2008). DUSP6 (MKP3) null mice show enhanced ERK1/2 phosphorylation at baseline and increased myocyte proliferation in the heart affecting disease susceptibility. J. Biol. Chem. *283*, 31246–31255.

45. Peirs, S., Matthijssens, F., Goossens, S., Van de Walle, I., Ruggero, K., de Bock, C.E., Degryse, S., Canté-Barrett, K., Briot, D., Clappier, E., et al. (2014). ABT-199 mediated inhibition of BCL-2 as a novel therapeutic strategy in T-cell acute lymphoblastic leukemia. Blood *124*, 3738–3747.

46. Chang, J.E., Medlin, S.C., Kahl, B.S., Longo, W.L., Williams, E.C., Lionberger, J., Kim, K., Kim, J., Esterberg, E., and Juckett, M.B. (2008). Augmented and standard

Berlin-Frankfurt-Munster chemotherapy for treatment of adult acute lymphoblastic leukemia. Leuk. Lymphoma *49*, 2298–2307.

47. Farber, C.R. (2010). Identification of a gene module associated with BMD through the integration of network analysis and genome-wide association data. J. Bone Miner. Res. *25*, 2359–2367.

48. Ritchie, M.E., Phipson, B., Wu, D., Hu, Y., Law, C.W., Shi, W., and Smyth, G.K. (2015). limma powers differential expression analyses for RNA-sequencing and microarray studies. Nucleic Acids Res. *43*, e47.

49. Zhang, H.-M., Kuang, S., Xiong, X., Gao, T., Liu, C., and Guo, A.-Y. (2015). Transcription factor and microRNA co-regulatory loops: important regulatory motifs in biological processes and diseases. Brief. Bioinform. *16*, 45–58.

50. Dweep, H., and Gretz, N. (2015). miRWalk2.0: a comprehensive atlas of microRNA-target interactions. Nat. Methods *12*, 697.

51. Zhang, H.-M., Liu, T., Liu, C.-J., Song, S., Zhang, X., Liu, W., Jia, H., Xue, Y., and Guo, A.Y. (2015). AnimalTFDB 2.0: a resource for expression, prediction and functional study of animal transcription factors. Nucleic Acids Res. *43*, D76–D81.

52. Zhang, H.-M., Chen, H., Liu, W., Liu, H., Gong, J., Wang, H., and Guo, A.Y. (2012). AnimalTFDB: a comprehensive animal transcription factor database. Nucleic Acids Res. *40*, D144–D149.

53. Dennis, G., Jr., Sherman, B.T., Hosack, D.A., Yang, J., Gao, W., Lane, H.C., and Lempicki, R.A. (2003). DAVID: Database for Annotation, Visualization, and Integrated Discovery. Genome Biol. *4*, 3.

54. Vlachos, I.S., Zagganas, K., Paraskevopoulou, M.D., Georgakilas, G., Karagkouni, D., Vergoulis, T., Dalamagas, T., and Hatzigeorgiou, A.G. (2015). DIANA-miRPath v3.0: deciphering microRNA function with experimental support. Nucleic Acids Res. *43* (W1), W460–W466.

55. Bindea, G., Mlecnik, B., Hackl, H., Charoentong, P., Tosolini, M., Kirilovsky, A., Fridman, W.H., Pagès, F., Trajanoski, Z., and Galon, J. (2009). ClueGO: a Cytoscape plug-in to decipher functionally grouped gene ontology and pathway annotation networks. Bioinformatics *25*, 1091–1093.

56. Assenov, Y., Ramírez, F., Schelhorn, S.-E., Lengauer, T., and Albrecht, M. (2008). Computing topological parameters of biological networks. Bioinformatics *24*, 282–284.

OMTN, Volume *12*

## Supplemental Information

# Differential Co-expression and Regulatory Network Analysis Uncover the Relapse Factor and Mechanism of T Cell Acute Leukemia

**Mei Luo, Qiong Zhang, Mengxuan Xia, Feifei Hu, Zhaowu Ma, Zehua Chen, and An-Yuan Guo**

## Supplemental Figures:

**Figure S1 The miRNA-TF-gene regulatory network for relapse/remission gene sets.**

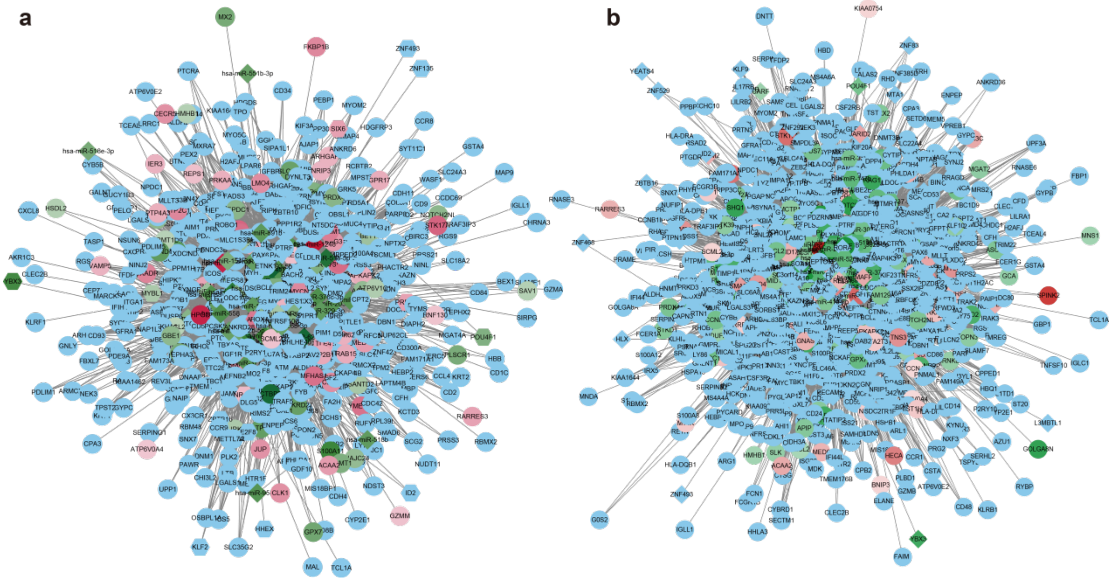


**Figure S1 The miRNA-TF-gene regulatory network for relapse/remission gene sets.** Hexagon: miRNA; Diamond: transcriptional factor; Ellipse: gene. The red shows up-regulation in relapse, the green represents down-regulation and the blue represents non-DEGs but co-expression. a) The miRNA-TF-gene regulatory network for relapse gene sets. b) The miRNA-TF-gene regulatory network for remission gene sets.

**Figure S2 The miRNA-TF-gene regulatory network for DEGs in relapse.**

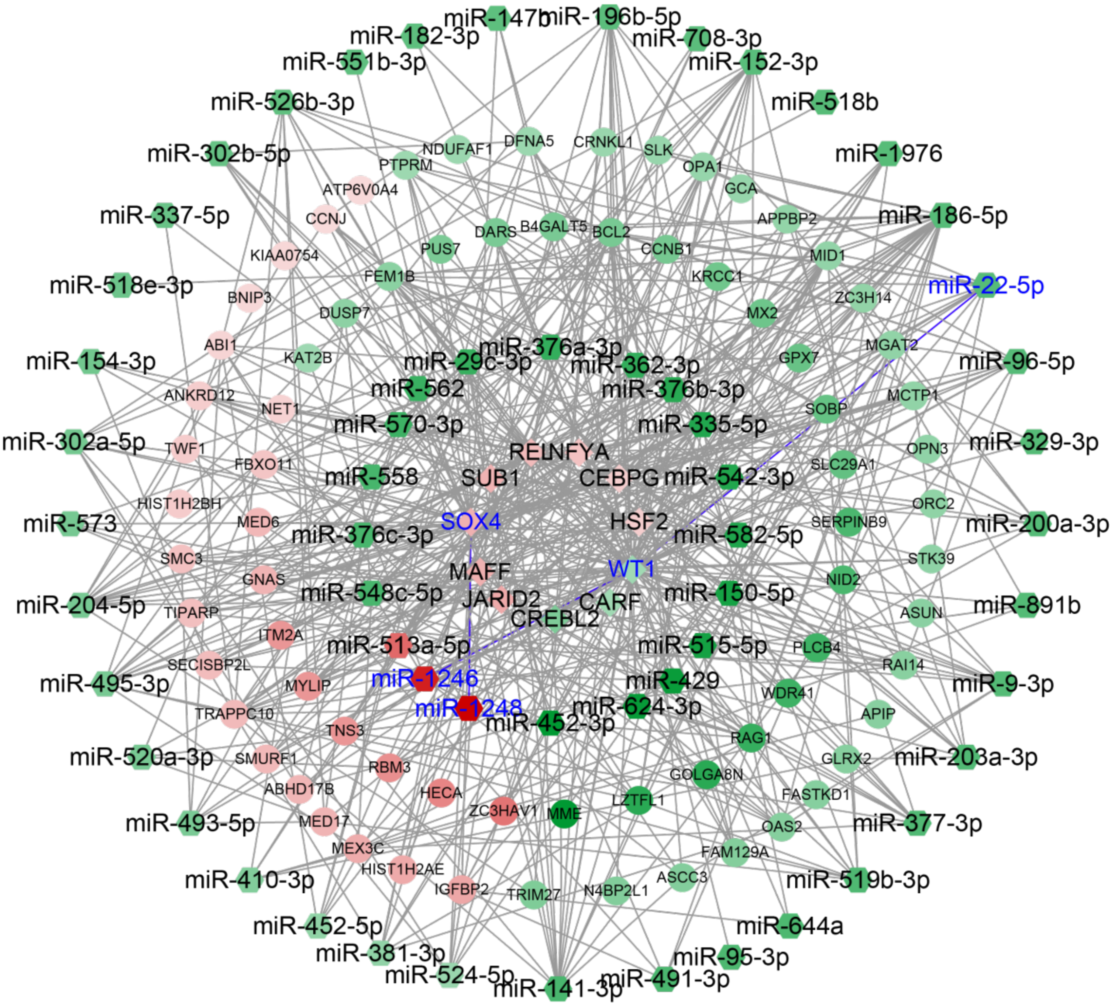


**Figure S2 The miRNA-TF-gene regulatory network for DEGs in relapse.** Hexagon: miRNA; Diamond: transcriptional factor; Ellipse: gene. The red shows up-regulation in relapse, the green represents down-regulation.

**Figure S3 DFS analysis of MFHAS1 and the optimization NOTCH2_siRNA.**

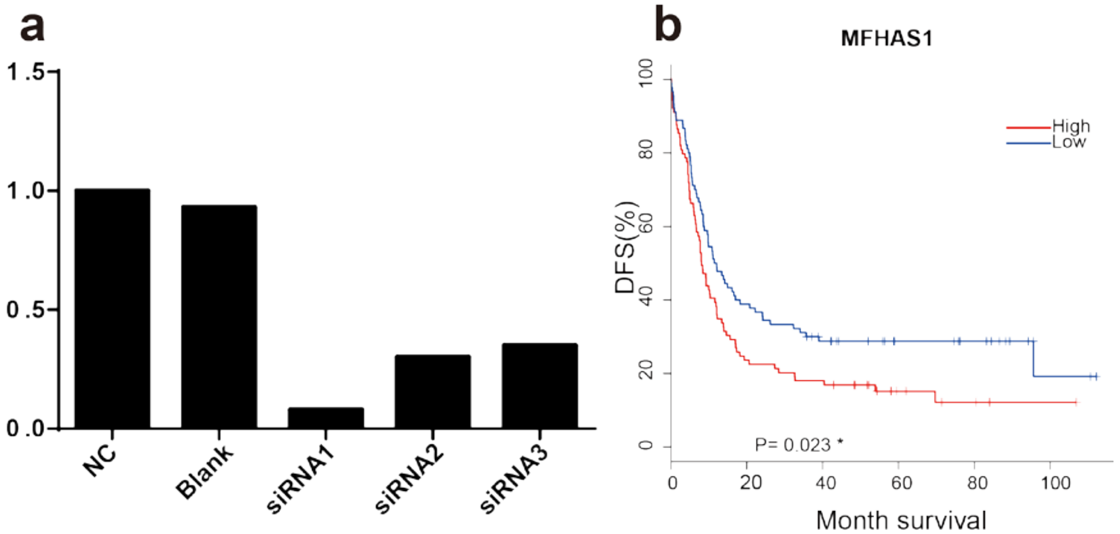


**Figure S3 DFS analysis of MFHAS1 and the optimization NOTCH2_siRNA.** a) The expression of NOCTH2 transfection with NOTCH2_siRNAs. b) Disease-free survival (DFS) curve of *MFHAS1* in LAML TCGA cohort.
